# Case Report: Arthroscopic “Debridement Antibiotics and Implant Retention” With Local Injection of Personalized Phage Therapy to Salvage a Relapsing *Pseudomonas Aeruginosa* Prosthetic Knee Infection

**DOI:** 10.3389/fmed.2021.569159

**Published:** 2021-05-05

**Authors:** Tristan Ferry, Camille Kolenda, Cécile Batailler, Romain Gaillard, Claude-Alexandre Gustave, Sébastien Lustig, Cindy Fevre, Charlotte Petitjean, Gilles Leboucher, Frédéric Laurent, The Lyon BJI Study group

**Affiliations:** ^1^Service des Maladies Infectieuses et Tropicales, Hôpital de la Croix-Rousse, Hospices Civils de Lyon, Lyon, France; ^2^Université Claude Bernard Lyon 1, Lyon, France; ^3^Centre interrégional de Référence pour la prise en charge des Infections Ostéo-Articulaires complexes (CRIOAc Lyon), Hospices Civils de Lyon, Lyon, France; ^4^Centre International de Recherche en Infectiologie, Inserm U1111, Université Claude Bernard Lyon 1, Ecole Normale Supérieure de Lyon, Lyon, France; ^5^Institut des Agents Infectieux, Laboratoire de bactériologie, Centre National de référence des staphylocoques, Hôpital de la Croix-Rousse, Hospices Civils de Lyon, Lyon, France; ^6^Service de Chirurgie Orthopédique, Hôpital de la Croix-Rousse, Hospices Civils de Lyon, Lyon, France; ^7^Pherecydes Pharma, Romainville, France; ^8^Service de Pharmacie Hospitalière, Hôpital de la Croix-Rousse, Hospices Civils de Lyon, Lyon, France

**Keywords:** bacteriophages, phage therapy, prosthetic-joint infection, *P aeruginosa*, phagotherapy

## Abstract

Bacteriophages are viruses that specifically target bacteria. They are considered to have a high potential in patients with prosthetic joint infection (PJI), as they have a synergistic anti-biofilm activity with antibiotics. We report here the case of an 88-year-old man (63 kg) with relapsing *Pseudomonas aeruginosa* prosthetic knee infection. The patient had severe alteration of the general status and was bedridden with congestive heart failure. As prosthesis explantation and/or exchange was not feasible, we proposed to this patient the use of phage therapy to try to control the disease in accordance with the local ethics committee and the French National Agency for Medicines and Health Products Safety (ANSM). Three phages, targeting *P. aeruginosa*, were selected based on their lytic activity on the patient's strain (phagogram). Hospital pharmacist mixed extemporaneously the active phages (initial concentration 1 ml of 1 × 10^10^ PFU/ml for each phage) to obtain a cocktail of phages in a suspension form (final dilution 1 × 10^9^ PFU/ml for both phages). Conventional arthroscopy was performed and 30 cc of the magistral preparation was injected through the arthroscope (PhagoDAIR procedure). The patient received intravenous ceftazidime and then oral ciprofloxacin as suppressive antimicrobial therapy. Under this treatment, the patient rapidly improved with disappearance of signs of heart failure and pain of the left knee. During the follow-up of 1 year, the local status of the left knee was normal, and its motion and walking were unpainful. The present case suggests that the PhagoDAIR procedure by arthroscopy has the potential to be used as salvage therapy for patients with *P. aeruginosa* relapsing PJI, in combination with suppressive antimicrobial therapy. A Phase II clinical study deserves to be performed to confirm this hypothesis.

## Case Report

**Figure 1:**
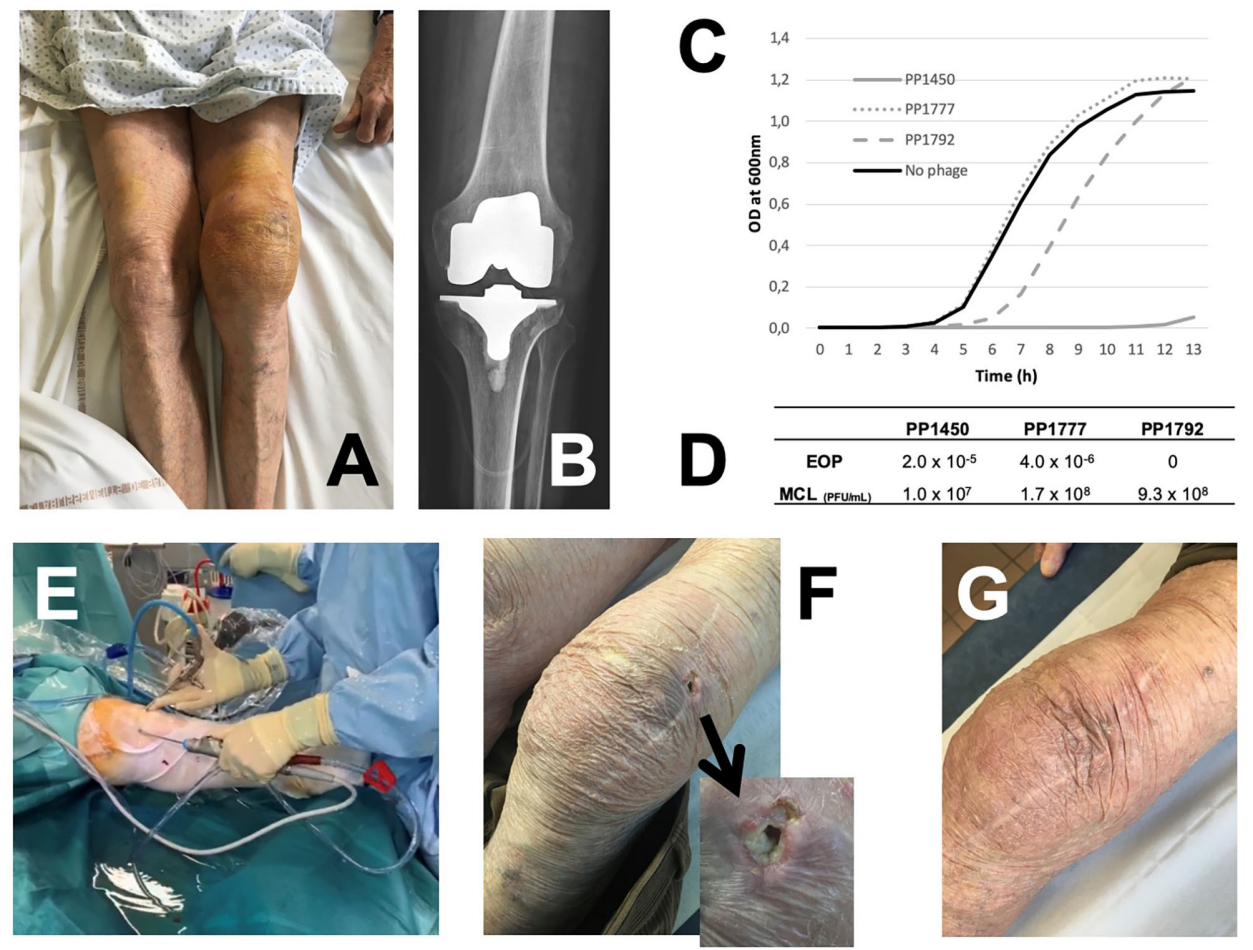
**(A)** Left knee joint effusion due to relapsing *P. aeruginosa* prosthesis knee infection; **(B)** X-ray showing no prosthesis loosening. The susceptibility of the patient's strain to the bacteriophages PP1450, PP1777, and PP1792 (phagogram) was performed using two complementary techniques: **(C)** For the kinetic assay, phages were incubated at a theorical multiplicity of infection (MOI, ratio of phages/bacteria) equal to 100 with the patient's strain. PP1450 was able to inhibit the bacterial growth (gray full line); PP1792 delayed the bacterial growth (gray dotted line) and PP1777 had no impact (gray dashed line). **(D)** For the plaque assay, titers obtained with the patient's strain and the reference strain are determined to calculate the efficiency of plating score (EOP) score (the closer to 1 is the score, the more efficient the phage is). Phages PP1450 and PP1777 were active on the patient's strain with an EOP score of 2.0 × 10^−5^ and 4.0 × 10^−6^, respectively. Partial lysis without PFU were observed for PP1792 (considered to have a weak bactericidal or bacteriostatic activity in this assay). **(E)** Arthroscopic DAIR with administration of the phage cocktail at the end of the procedure through the arthroscope. **(F)** Ulceration of a subcutaneous nodule on the external side of the knee observed 2 months after the arthroscopy. **(G)** Finally, a favorable outcome under suppressive antimicrobial therapy.

An 88-year-old man (63 kg) had a past history of arrhythmia with severe cardiomyopathy and bilateral arthroplasties several years ago. A colonoscopy was performed and was followed a few days later by clinical signs of septic arthritis of the left knee. The patient did not have fever, but CRP was ~200 mg/L. Echocardiography disclosed no signs of endocarditis. Analysis of joint puncture showed infiltration by polymorphonuclear cells (57,000/mm^3^) and *Pseudomonas aeruginosa* susceptible to ceftazidime and ciprofloxacin grew in culture. Open (i.e., by arthrotomy) Debridement Antibiotics and Implant Retention (DAIR) procedure was performed (1), followed by treatment with intravenous ceftazidime 6 g/day plus oral ciprofloxacin (500 mg bid). Three weeks after the surgery, the outcome seemed to be favorable, ceftazidime was stopped, and ciprofloxacin was prolonged for a total duration of 12 weeks. Six months later, the patient experienced a relapse of the joint knee effusion (Figure 1A), with heart failure. CRP was ~100 mg/L. X-ray disclosed no loosening of the prosthesis (Figure 1B). A knee joint puncture showed *P. aeruginosa* persistence, with the same antimicrobial susceptibility profile. The patient was totally bedridden with severe alteration of the general status. As general anesthesia was contraindicated to explant the prosthesis or to perform a new open DAIR, we proposed to this patient the use of phage therapy to try to control the disease. After multidisciplinary meetings in our reference center (which is certified by the French ministry of health for the management of complex bone and joint infection), (2) and in accordance with the local ethics committee, this case was individually discussed with the French National Agency for Medicines and Health Products Safety (ANSM), to validate that no other options could be proposed without excessive risk of death. Phages, targeting *P. aeruginosa*, were selected from the Pherecydes Pharma library based on their lytic activity on the patient's strain (3). The phages have been produced in a non-GMP facility but have undergone a thorough quality evaluation with multiple quality control tests. Phagograms were performed using kinetic assay and the plaque assay, to calculate the efficiency of plating score (EOP) as previously described (Figures 1C,D) (4). Three bacteriophages (PP1450, PP1777, and PP1792) were selected, as they were totally or partially active for at least one technique. PP1450 and PP1777 belong to the *Myoviridae* family, and their closest relative in public database (Genbank) belong to the *Pbunavirus* genus (ICTV 2018). PP1792 belongs to the *Podoviridae* family and *Bruynoghevirus* genus. The patient signed a written consent, explaining the procedure and the risk/benefit ratio. Hospital pharmacist mixed extemporaneously the active phages [initial concentrations 1 ml of 1 × 10 (5) PFU/ml for each phage] to obtain a cocktail of phages in a suspension form [final concentration of 1 × 10 (6) PFU/ml for both phages]. Conventional arthroscopy was performed (Figure 1E) using anteromedial and anterolateral entry points and washing of joint with saline. After drainage of the arthroscopic liquid, 30 cc of the phage suspension was injected through the arthroscope. Then, entry points were closed to be waterproof. No other bacteria grew in culture. The patient received again 3 weeks of intravenous ceftazidime (6 g/day) and oral ciprofloxacin (500 mg bid). The patient rapidly improved with disappearance of signs of heart failure and pain of the left knee (Supplementary Video 1). The CRP reached normal values quickly. A subcutaneous nodule that has spontaneously ulcerated appeared on the external side of the knee (Figure 1F), without discharge or any communication with the joint, and then disappeared spontaneously. At 6 months, the local status of the left knee was normal (Figure 1G) and its motion and walking were unpainful (Supplementary Videos 2, 3). The dose of ciprofloxacin was reduced to 250 mg bid as suppressive antimicrobial therapy to prolong the remission of symptoms (7). One year after the phage administration, the patient unfortunately died from lithiasic pancreatitis, without any clinical signs of prosthetic joint infection (PJI).

Bacteriophages are viruses that specifically target bacteria. They are considered to have a high potential in patients with PJI, as they have a synergistic anti-biofilm activity with antibiotics (8, 9). In several patients with relapsing chronic PJI due to *S. aureus* for whom explantation was not Possible, we already performed open DAIR and used selected bacteriophages that were injected into the joint (PhagoDAIR procedure) with a good clinical response (6, 10). Moreover, recent data from animal models provided further support for phage therapy as effective adjunctive treatment for PJI (5). In the present case, arthroscopic DAIR was the only possible surgery, to limit the risk of perioperative death, whereas this procedure is considered to have no place in the management of PJI due to (i) an incomplete debridement (peroperative dislocation is not feasible), (ii) an inability to exchange the polyethylene part of the prosthesis, and (iii) an extremely low success rate. In counterpart, it is easy to inject into the joint the bacteriophages preparation during arthroscopy, and the joint remained perfectly tight (6). The opportunity to target the biofilm is a potential key determinant in such patients if the prosthesis cannot be explanted. By using personalized phage therapy as adjuvant therapy, the aim is to act locally on bacteria embedded in biofilm sticked on the implant surface into the joint cavity, as demonstrated recently in animal and *in vitro* models (11).

This case report leads to question the intrinsic capacity of the phage therapy to improve the outcome of the patient, as he was also managed with surgery and antibiotics. However, as the patient presented relapsing PJI after previous standard of care treatments, the expected success rate of iterative DAIR procedure performed by arthroscopy and followed by suppressive antimicrobial therapy was very limited if the bacteriophages had no effect on the biofilm. Indeed, arthroscopic DAIR is usually contraindicated in patients with PJI, as (i) the risk of relapse is particularly high if the polyethylene part cannot be changed, likely because such plastic surface promotes biofilm formation; (ii) the reduction of the bacterial load is significantly lower in comparison with open DAIR; and (iii) the evidence and guidelines discourage its use as too much worse outcomes were reported (1, 7, 11–16). Finally here, we hypothesized that the phage administration has helped the suppressive antimicrobial therapy to succeed in the control of the infection, i.e., to prolong the remission (15, 16).

The present data suggest that the PhagoDAIR procedure by arthroscopy has the potential to be used as salvage therapy for patients with *P. aeruginosa* relapsing PJI, in combination with suppressive antimicrobial therapy. A Phase II clinical study deserves to be performed to confirm this hypothesis.

## Data Availability Statement

The raw data supporting the conclusions of this article will be made available by the authors, without undue reservation.

## Ethics Statement

The studies involving human participants were reviewed and approved by Hospices Civils de Lyon Ethic Committee. The patients/participants provided their written informed consent to participate in this study. Written informed consent was obtained from the individual(s) for the publication of any potentially identifiable images or data included in this article.

## Author Contributions

TF managed the patients and coordinated the treatment procedure. TF wrote the draft of the manuscript. CB, RG, and SL participated to the patient care. CK, C-AG, CF, and CP participated to the microbiological work. GL prepared the phage mix. All authors contributed to the article and approved the submitted version.

## Conflict of Interest

CF and CP are employed by Pherecydes Pharma. The remaining authors declare that the research was conducted in the absence of any commercial or financial relationships that could be construed as a potential conflict of interest.

## Supplementary Material

Supplementary Video 1 Video showing the patient walking without any pain 3 weeks after the surgery.

Supplementary Video 2 Video showing the motion of the left knee without any pain 3 months after the surgery.

Supplementary Video 3 Video showing the patient walking without any pain 3 months after the surgery.
